# Use of caffeine in 19,660 randomly selected U.S. adults: the role of overweight and obesity

**DOI:** 10.3389/fnut.2025.1588447

**Published:** 2025-06-25

**Authors:** Larry A. Tucker, Frank Beltran

**Affiliations:** Department of Exercise Sciences, College of Life Sciences, Brigham Young University, Provo, UT, United States

**Keywords:** appetite suppressant, stimulant, NHANES, BMI, waist, abdominal fat

## Abstract

**Background:**

Caffeine is a stimulant. It is one of the most consumed drugs in the United States. The present investigation was conducted to determine the relationships between BMI and waist circumference, and caffeine intake. The specific objective was to determine whether adults with higher BMIs or larger waist circumferences consume more or less caffeine than their counterparts.

**Methods:**

A sample of 19,660 randomly selected women and men, 20–75 years old, representing the U.S. adult population, was studied using a cross-sectional design. Data was collected as part of the National Health and Nutrition Examination Survey (NHANES) from 2009 through 2018. A number of covariates were controlled statistically to minimize their influence on the results: age, gender, race, year of assessment, alcohol use, smoking, time spent in moderate to vigorous physical activity, total energy intake (kcal), and consumption (g per 1,000 kcal) of dietary fiber, carbohydrate, protein, fat, sugar, and saturated fat.

**Results:**

After adjusting for all the covariates, results showed that in U.S. men, there was a dose-response relationship between higher BMI categories and higher caffeine consumption (*F* = 4.1, *p* = 0.0092). After adjusting for all the covariates, the relationship between waist circumference and caffeine intake in men was linear (*F* = 8.0, *p* = 0.0060). In U.S. women, after adjusting for all the covariates, there was a weaker but significant relationship between the BMI categories and caffeine intake (*F* = 3.4, *p* = 0.0232). In women, the association between waist circumference and caffeine intake was not linear (*F* = 0.0, *p* = 0.8490), but was quadratic (*F* = 18.9, *p* < 0.0001) with all the covariates controlled.

**Conclusions:**

Overall, this study found that U.S. men with larger body mass and waist levels consumed higher amounts of caffeine than their counterparts. U.S. women did also, but the relationships were quadratic, not linear like the men's. It appears that the drug, caffeine, has multiple properties that appeal to adults with higher BMI and waist circumference levels. Additional research is needed to better understand why adults with larger body mass and waist sizes consume significantly more caffeine than their counterparts.

## Introduction

Caffeine is a simulant. It is one of the most consumed drugs in the United States with reports showing that 89% of Americas ingest caffeine on a daily basis (1). Such frequent use can be accredited to caffeine's presence in a variety of commonly consumed items. Coffee, tea, chocolate, energy drinks, and supplements are among the many sources that contain caffeine (2). The justification for caffeine being consumed by a large portion of the population is because of its ability to improve awareness and temporarily reduce fatigue while also boosting performance with work that requires heightened cognitive functions (3).

Caffeine is not only known for its stimulating properties, which include increased alertness and energy levels (4), but also for its potential for affecting metabolism and appetite. Numerous studies have demonstrated this effect. In a double-blind study by Carter et al. (5) participants received one of four conditions and found that the beverage containing caffeine led to the greatest decrease in appetite and energy intake. Another study showed that caffeine further intensified the appetite-suppressant qualities of another stimulant, nicotine, further demonstrating caffeine's effect on decreasing energy intake (6).

Because caffeine decreases appetite, it is studied as a viable aid in weight loss, especially for those who have overweight or obesity. For example, Westerterp–Plantenga et al. (7) conducted an experiment where participants were placed in a group that either consumed low or high amounts of caffeine. They found that those who consumed higher amounts of caffeine lost more weight than those consuming less caffeine. Furthermore, a meta-analysis conducted by Tabrizi et al. (8) reviewed multiple randomized controlled trials and concluded that the consumption of caffeine tends to reduce weight, BMI, and body fat. Such findings provide support for the use of the drug, caffeine, for those who desire to decrease energy consumption and reduce body weight.

In 2013, the American Medical Association classified obesity as a disease (9). Obesity, or the excessive accumulation of body fat, is linked to various serious diseases such as hypertension, cardiovascular disease, certain cancers, and more (10). These chronic diseases are some of the most common when it comes to preventable, premature death, and obesity rates are only increasing. Obesity prevalence in the United States in 2000 was 30.5% and in 2020, it was 41.9% (11).

Given the established effects of caffeine consumption, including appetite and energy intake suppression and weight loss aid, do adults with overweight or obesity turn to caffeine more or less than others? In the natural environment, where adults govern their own intake of caffeine and food, do those with excess body weight use caffeine more than their peers? To date, this research question has rarely, if ever, been addressed. Moreover, an investigation designed to study the extent that individuals with overweight or obesity consume higher or lower amounts of caffeine than others has never been performed using a large random sample representative of the U.S. adult population. Consequently, the present investigation was conducted.

The primary objective was to study the innate relationship between different levels of body mass and consumption of caffeine in a random sample of almost 20,000 U.S. adults. In other words, in the natural environment where adults govern their own intake of caffeine and food, do adults with excess body weight use caffeine more or less than their peers? Another core objective was to determine if adults with abdominal obesity consume more or less caffeine than their counterparts. A secondary aim was to ascertain the influence of several potential confounding variables, including age, gender, race, alcohol use, smoking, dietary fiber intake, total energy intake, time spent engaged in moderate to vigorous physical activity, and numerous other dietary intake variables, on the key relationships.

## Methods

### Design

Data from the National Health and Nutrition Examination Survey (NHANES) was used to complete this cross-sectional study. NHANES is a government sponsored program under the direction of the Centers for Disease Control and Prevention (CDC) that started in the 1960's. NHANES uses both consultations and medical examinations to evaluate the overall health of people living in the United States. According to NHANES, “Health interviews are conducted in respondents' homes. Health measurements are performed in specially designed and equipped mobile centers” (12). Data from NHANES is collected in 2-year cycles. The present study utilized information from five NHANES cycles spanning from 2009 to 2018.

The files posted online by NHANES contained no confidential information. In short, no data can be connected to a specific individual. Each participant provided written consent to take part in the national survey (13). The Ethics Review Board (ERB) of the National Center for Health Statistics (USA) approved the NHANES data collection protocol. The ethical approval codes for NHANES data collection from 2009 to 2018 were: continuation of protocol #2005-06, #2011-17, and #2018-01, as shown on the NHANES website: https://www.cdc.gov/nchs/nhanes/about/erb.html?CDC_AAref_Val=https://www.cdc.gov/nchs/nhanes/irba98.htm

### Sample

Participants were randomly selected using a complex multistage sampling strategy. Therefore, the sample was representative of the U.S. adult population (12). The procedure for determining where to collect data was as follows: First, NHANES randomly selected counties or groups of small counties. Second, roads or blocks containing multiple residences were randomly chosen. Third, households were randomly selected. Lastly, individuals from those households were randomly chosen (14).

A total of 37,251 women, men, and children, ages 1–80+ years were selected by NHANES from 2009 to 2018. With the sample delimited to adults 20–75 years old, there were 20,120 participants. There were 38 women who were pregnant reducing the sample to 20,082. Adults who fasted or reported extremely low energy intakes (<400 kcal per day) for one or both 24-h dietary recall assessments were not included in the sample (*n* = 273), leaving 19,809 participants. There were 149 adults with missing BMI values reducing the sample to 19,660 for analyses focused on BMI, and 418 had missing data for their waist circumference, resulting in a sample of 19,242 for analyses including waist size.

### Instrumentation and measurement methods

Information was obtained on 16 variables: total caffeine intake, body mass index (BMI), waist circumference, age, sex, race, alcohol use, smoking, total energy intake, time spent engaged in moderate to vigorous physical activity (MVPA), fiber consumption per 1,000 kcal, and intake of carbohydrate, protein, fat, sugar, and saturated fat per 1,000 kcal.

### Diet

For diet, all information was recorded via two 24-h recall interviews. The first interview was conducted in person and the participants recalled what they ate from midnight to midnight with guidance from the interviewer. The second interview occurred through a telephone interview three to 10 days later and again, participants recalled what they ate from midnight to midnight. Participants had numerous measuring guides to assist them in the first interview. They were then given measuring guides to take home to ensure their recollection was as accurate as possible for the second interview as well (15).

Those who conducted the 24-h recall interviews underwent careful selection, preparation, and training to ensure that the data collected was as accurate as possible. Each of these individuals completed a degree in nutrition or a similar focus. They also had to be bilingual. Before conducting interviews, each interviewer underwent a 1-week training course where practice interviews were performed under supervision to evaluate how well they gathered data. The training sessions were performed every year to remind interviewers of the correct steps and procedures in collecting and recording data. During the interviews, interviewers followed scripted guidelines and used a computer program to ensure that all interviews followed the same format (15, 16).

### Caffeine intake

Consumption of total dietary caffeine was measured in milligrams per day after considering all the foods that the participants reported in their two 24-h recall interviews. The average caffeine intake derived from the two recalls was used. This included coffee, tea, energy drinks, chocolate, and other consumables. The amount of caffeine consumed per day was estimated using the Food and Nutrient Database for Dietary Studies (FNDDS) from the United States Department of Agriculture (USDA).

### Body mass index (BMI)

When collecting measurements for BMI, NHANES took the participants weight in kilograms and then divided it by their height in meters squared. The results were then rounded to one decimal place allowing the participants to be placed into different categories, including underweight (BMI: <18.5), normal weight (BMI: 18.5–24.9), overweight (BMI: 25.0–29.9), or obese (BMI: **≥**30.0).

### Waist circumference

Participants were taken to a designated room that had wall mirrors and other equipment designed to help with the measurement procedure. A trained technician instructed the participants to fold their arms so that hands were touching opposite shoulders, and their top clothing was clipped at the front to expose the midsection. The technician then stood on the individual's right side and located the right ilium. A mark was made at the top of the ilium and then the technician extended the measuring tape around the waist at the level of the mark. The technician verbally reported the waist circumference, and another staff member recorded the measurement. Throughout the process, both staff members ensured that the tape was applying minimal pressure and was taken after the participant exhaled normally. The tape also had to be parallel to the floor and at the level of the mark made at the ilium so the measurement would be as accurate as possible (17).

### Covariates

Age was self-reported and NHANES defined sex as the sex assigned at birth. NHANES categorized race/ethnicity into five separate categories. These categories were as follows: Mexican American, Other Hispanic, Non-Hispanic White, Non-Hispanic Black, and Other Race which included multi-racial.

A total of 10 years of data were used in this study. Data were collected during five 2-year cycles: 2009–2010, 2011–2012, 2013–2014, 2015–2016, and 2017–2018. The 2019–2020 cycle was interrupted due to the outbreak of the COVID-19 virus. Hence, the last survey cycle employed was 2017–2018. A categorical variable with five levels for each of the 2-year survey cycles was included as a covariate in each statistical model.

NHANES categorized alcohol use into three separate categories based on the number of alcoholic drinks typically consumed on days that the participant drank during the past 12 months. These categories were as follows: abstainers, moderate drinkers, and heavy drinkers. Participants were categorized as abstainers if they reported drinking no alcohol in the past year. Male participants were categorized as moderate drinkers if they typically consumed one to two drinks on days that they drank, while female participants were categorized as moderate drinkers if they consumed one drink containing alcohol on days that they drank. Male participants were categorized as heavy drinkers if they consumed three or more alcoholic drinks on days that they drank, while female participants were categorized as heavy drinkers if they consumed two or more alcoholic drinks on days that they drank in the past year.

Dietary fiber intake was calculated through the foods and drinks that were reported in the two 24-h recall interviews and was reported in grams per 1,000 kcal. This included grams of insoluble fiber as well as grams of soluble fiber.

Total energy intake was calculated by using the foods and drinks that were reported in the two 24-h recall interviews and was recorded in kilocalories (kcal). Individuals who reported intaking <400 kcal in either or both of the 24-h interviews were not included in the sample. Grams of carbohydrate, sugar, protein, fat, and saturated fat were calculated per 1,000 kcal consumed using the average of the two 24-h recall assessments.

Participants were categorized into one of three smoking categories based on questions asking if they had ever smoked, if they currently smoked, if they smoked in the past, how long since they quit, the number of cigarettes they smoked currently or in the past, etc. Responses were used to categorize participants as non-smokers, current smokers, and ex-smokers.

To operationalize moderate to vigorous physical activity (MVPA), participants were asked a series of questions to determine the amount of time they spent engaged in activities that were moderate intensity and activities that were vigorous. For both categories, participants were asked if their work (paid or unpaid), yard work, chores, exercise, etc. involved moderate and/or vigorous intensity exertion requiring small (moderate intensity) and/or large (vigorous intensity) increases in breathing or heart rate for at least 10 min. They were then asked how many days per week they participated in moderate activity and then vigorous physical activity. Finally, participants were asked how much time they spent in moderate activity and then vigorous activity on those days. MVPA was indexed by summing the minutes of moderate activity per week and the minutes of vigorous activity per week for each participant.

### Data analysis

NHANES employs a sophisticated, multi-stage sampling procedure so the results can be generalized to the non-institutionalized adult population of the United States. For this to occur, strata, clusters, and individual sample weights were included with each statistical analysis.

Given the sample of the present investigation was in excess of 19,000 individuals, most would assume that statistical power was extremely high. However, this was not the case. The multi-level sampling strategy required that the number of strata (74) be subtracted from the number of clusters (152) resulting in a total of 78 degrees of freedom (df) in the denominator for each model. Hence, statistical power was not extreme in this study.

There were two exposure variables, body mass index (BMI) and waist circumference. The outcome variable was total caffeine intake. Age, race, year of assessment, alcohol consumption, smoking behavior, moderate to vigorous physical activity (MVPA), average energy intake, fiber consumption per 1,000 kcal, and intake of carbohydrate, protein, fat, sugar, and saturated fat per 1,000 kcal were employed as the covariates. Because there were significant differences in caffeine consumption, BMI, and waist circumference between females and males, analyses were conducted separately for women and men.

The SAS SurveyFreq procedure was used to produce frequency results and standard errors for each level of the categorical variables. SAS SurveyMeans was employed to generate means and standard errors for the continuous variables. SAS SurveyReg was utilized to determine mean caffeine intake differences across the categorical variables of BMI and waist size. SurveyReg was also used to evaluate mean energy intake (kcal) differences across caffeine intake tertiles. The *F*-statistic was used to determine the extent that the variance between the groups was statistically greater than the variance within the groups. Partial correlation and the LSMeans technique were used to generate adjusted means and to determine statistical differences across the mean caffeine scores.

The SAS SurveyReg procedure was also employed to measure the extent of the linear relationship between BMI and caffeine consumption, and between waist circumference and caffeine intake, with both measures treated as continuous variables. To determine the extent that associations were quadradic, BMI-squared (BMI^2^) was included in the model following the BMI linear term. The same protocol was employed for the waist circumference and caffeine intake association. Partial correlation was used to adjust for differences in the covariates.

SAS version 9.4 (SAS Institute, Inc., Cary, NC) was utilized to measure the key associations. Alpha was set at the 0.05 level to affirm statistical significance. The tests were all two-sided.

## Results

Data collection spanned 10 years from 2009 to 2018. This sample had a mean (±SE) age of 45 ± 0.3 years, and the average consumption of caffeine (±SE) was 166 ± 3.4 mg/day. Mean (±SE) BMI was 29.3 ± 0.1 kg/m^2^ and mean (±SE) waist circumference was 99.6 ± 0.3 cm. The average consumption of energy (±SE) was 2,124 ± 9.6 kcal/day. Table 1 shows the different percentiles for the main continuous variables of this study.

**Table 1:** Percentile distributions of the continuous variables representing U.S. adults (*n* = 19,660).

**Variable**	**Percentile**				
	**10th**	**25th**	**50th**	**75th**	**90th**
Caffeine intake (mg/day)	2.6	39.7	122.4	237.0	368.4
Women (*n* = 10,282)	2.1	34.1	105.9	205.4	333.8
Men (*n* = 9,378)	3.5	47.3	142.7	265.0	400.9
Fiber intake (g/1,000 kcal)	4.4	5.8	7.8	10.4	13.5
Women	4.8	6.2	8.3	11.0	14.2
Men	4.2	5.5	7.3	9.9	12.6
Carb. intake (g/1,000 kcal)	89.6	104.1	119.5	134.6	149.0
Women	92.1	106.7	121.7	136.5	150.6
Men	86.8	101.7	116.9	132.3	147.0
Protein intake (g/1,000 kcal)	28.3	33.0	38.8	45.7	53.8
Women	28.1	32.6	38.4	45.5	53.4
Men	28.6	33.4	39.1	45.8	54.2
Fat intake (g/1,000 kcal)	28.0	33.1	38.4	43.7	48.7
Women	28.1	33.2	38.4	43.9	48.9
Men	27.9	33.0	38.4	43.5	48.4
Sugar intake (g/1,000 kcal)	25.9	35.9	49.2	64.0	79.5
Women	28.2	38.1	51.0	65.9	81.6
Men	23.5	33.8	47.2	62.0	76.9
Saturated fat (g/1,000 kcal)	8.0	10.0	12.3	14.6	17.0
Women	8.0	9.9	12.2	14.5	16.9
Men	8.1	10.1	12.3	14.7	17.1
Energy intake (kcal/day)	1,224	1,561	2,000	2,551	3,160
Women	1,096	1,382	1,742	2,147	2,598
Men	1,485	1,865	2,341	2,919	3,536
MVPA (min/day)	0	0	28.4	60.0	126.9
Women	0	0	19.7	57.8	116.2
Men	0	0	29.3	89.4	173.8
Age (years)	24.0	31.6	45.1	57.4	66.1
Women	24.2	32.1	45.6	57.7	66.6
Men	23.8	31.2	44.7	57.2	65.7
Body mass index (kg/m^2^)	21.6	24.3	28.2	33.0	38.6
Women	21.1	23.8	28.2	33.7	39.9
Men	22.4	24.9	28.2	32.4	36.9
Waist circumference (cm)	79.0	87.4	98.0	109.6	121.6
Women	76.6	84.4	95.4	107.7	120.4
Men	82.6	90.8	100.7	111.0	122.8

Table values included person-level weighted adjustments based on the sampling methods, so values represent those of the U.S. adult population. For each continuous variable, the first line shows the combined results, women and men together, followed by results for women only and men only. Carb. intake, carbohydrate intake; MVPA, moderate to vigorous physical activity.

Table 1 shows that 50% (±SE) of U.S. adults consumed about 122.4 ± 3.3 mg of caffeine per day with the top 10% (±SE) reporting 368.4 ± 7.0 mg per day. Moderate to vigorous physical activity showed a median value (±SE) of 28.4 ± 2.4 min per day. The least active adults, the bottom quartile, reported that they did not engage in any MVPA. Fiber intake was also low with a median (±SE) of 7.8 g/1,000 ± 0.1 kcal/day. The median (±SE) for BMI was 28.2 ± 0.1 kg/m^2^ indicating that 50% of the sample had BMI levels between the overweight and obese cut-points.

Table 2 presents an overview of the characteristics of the sample with regards to the categorical variables. The sample included about 6% more women than men, consistent with the U.S. census. Each 2-year cycle had about the same number of adults, roughly 20%. About 57% of the sample reported that they drank alcohol regularly, and 78% were non-smokers. Over 70% of the sample was found to have overweight or obesity.

**Table 2:** Characteristics of the sample based on the categorical variables (*n* = 19,660).

**Categorical variable**	***N***	**%**	**SE**
**Sex**			
Women	10,103	51.4	0.48
Men	9,557	48.6	0.48
**Race/ethnicity**			
Mexican American	1,790	9.1	0.88
Other Hispanic	1,201	6.1	0.56
Non-Hispanic White	12,675	64.5	1.57
Non-Hispanic Black	2,259	11.5	0.81
Other	1,735	8.8	0.50
**Year of assessment**			
2009–2010	3,799	19.3	0.95
2011–2012	3,860	19.6	1.06
2013–2014	3,955	20.1	1.08
2015–2016	3,997	20.3	0.98
2017–2018	4,049	20.6	0.81
**Smoking**			
Non-smoker	15,350	78.1	0.62
Ex-smoker	564	2.9	0.19
Current smoker	3,746	19.1	0.57
**Alcohol use**			
Abstainer	8,469	43.1	0.94
Moderate drinker	5,352	27.2	0.78
Heavy drinker	5,839	29.7	0.57
**Body mass index**	
Underweight	281	1.4	0.13
Normal weight	5,332	27.1	0.68
Overweight	6,308	32.1	0.67
Obese	7,739	39.4	0.75
**Abdominal obesity**			
No	8,261	42.9	0.82
Yes	10,981	57.1	0.82

SE refers to the standard error of the percentage. The *N* and % columns refer to the number of subjects and the percentage of participants, after the NHANES sample weights were applied. Weighted values are more meaningful than unweighted values because they can be generalized to the U.S. adult population. The cut-points for abdominal obesity were gender-based: women: 88 cm, men: 102 cm.

Table 3 shows the mean differences in caffeine intake across the traditional BMI categories (Underweight, Normal weight, Overweight, and Obese) displayed by sex. Adjustments were made statistically based on two models with different covariates. For model 1, adjustments were employed for differences in age, race, year of assessment, alcohol use, smoking, and minutes engaged in moderate to vigorous physical activity. For model 2, several dietary variables were added to model 1 as covariates, including total energy intake (kcal), and intake (g per 1,000 kcal) of carbohydrate, protein, fat, fiber, sugar, and saturated fat. As seen in Table 3, for U.S. men, mean differences in caffeine intake across the BMI categories were highly significant and dose-response for both models. For U.S. women, there was a significant but non-linear relationship between BMI and caffeine intake. For women and men, in general, those in the heavier BMI categories consumed larger amounts of caffeine than their counterparts. This was especially true for U.S. men.

**Table 3:** Mean differences in caffeine intake across categories of BMI, after adjusting for the covariates (*n* = 19,660).

**Body mass index**										
	**Underweight**		**Normal**		**Overweight**		**Obese**			
**Outcome variable**	**Mean**	**±SEM**	**Mean**	**±SEM**	**Mean**	**±SEM**	**Mean**	**±SEM**	***F***	***p*** **-value**
**Caffeine intake (mg)**	
**Model 1**	
Men	126.7^a^	16.5	169.2^b^	8.7	174.8^b^	8.8	186.6^c^	8.8	4.9	0.0034
Women	138.0^a^	12.7	151.8^a^	9.7	168.9^b^	10.3	157.4^a^	8.9	2.9	0.0416
**Model 2**	
Men	125.5^a^	18.8	171.9^b^	8.5	177.0^b^	8.6	186.7^b^	8.1	4.1	0.0092
Women	132.9^a^	12.2	149.4^a, b^	9.2	166.4^c^	9.7	154.3^b^	8.4	3.4	0.0232

SE, standard error of the mean. *F* is the *F*-statistic, the ratio of the between group variance over the within group variance. Means on the same row with the same superscript letter are not significantly different. Means have been adjusted based on the covariates. Model 1 included the demographic and lifestyle covariates: age, race, year of assessment, alcohol intake, smoking status, and time spent in moderate to vigorous physical activity. Model 2 included the same covariates as Model 1 and total energy intake (kcal), and intake of each of the following dietary factors (g per 1,000 kcal) fiber, carbohydrate, protein, fat, sugar, and saturated fat. There were 78 degrees of freedom in the denominator. For men, the caffeine differences between those with normal body weight and those with overweight or obesity were marginally significant (*p* = 0.06). For women (model 2), the caffeine difference between those with underweight and obesity was marginally significant (p = 0.08). The BMI categories were defined as follows: the underweight group had BMIs of <18.5, the normal weight group had BMIs ≥18.5 and <25.0. The overweight group had BMIs ≥25 and <30.0, and those in the obese category had BMIs of ≥30.

The linear association between BMI and caffeine intake was also evaluated with both variables treated as continuous measures. For men, after adjusting for all the covariates, the relationship between BMI and caffeine intake was linear (*F* = 6.4, *p* = 0.0135). The regression coefficient was 1.3 (SE: 0.5) signifying that caffeine consumption was 1.3 mg higher for each unit increase in BMI. For U.S. women, the relationship between caffeine intake and BMI was not linear (*F* = 0.0, *p* = 0.8490). However, with all the covariates controlled and the linear BMI term in the model ahead of BMI^2^, the quadratic form of BMI (BMI^2^) was strongly related to caffeine intake (*F* = 18.9, *p* < 0.0001). As seen in Table 3, where BMI was treated as a categorical variable, for women, caffeine intake increased as BMI levels increased, then caffeine consumption dipped when BMI levels reached their uppermost levels.

With waist size and caffeine intake both treated as continuous variables and all the covariates controlled, the relationship was linear and significant for U.S. men (*F* = 8.0, *p* = 0.0060). The quadratic relationship was not significant. In short, as waist size increased in men, caffeine intake also increased. For women, the relationship between waist size and caffeine consumption was not linear (*F* = 0.1, *p* = 0.8211), but was quadratic (*F* = 15.6, *p* = 0.0002), similar to the BMI and caffeine intake association in U.S. women.

Table 4 shows the mean differences in caffeine intake across the waist circumference categories, normal and abdominal obesity, displayed by sex. Adjustments were made statistically for differences in the demographic and lifestyle covariates (model 1) and for all the covariates (model 2), which also included the seven dietary covariates. For both women and men, those with abnormal waist circumferences consumed significantly higher amounts of caffeine than their counterparts.

**Table 4:** Mean differences in caffeine intake across categories of waist circumference, after adjusting for the covariates.

	**Waist circumference**					
	**Normal**		**Abdominal obesity**			
**Outcome**	**Mean**	**±SEM**	**Mean**	**±SEM**	***F***	***p*** **-value**
**Caffeine intake (mg)**						
**Model 1**						
Men	171.9^a^	8.1	187.1^b^	7.9	5.4	0.0226
Women	150.2^a^	10.1	163.0^b^	9.2	6.2	0.0148
**Model 2**						
Men	174.9^a^	7.6	186.9^b^	7.3	3.8	0.0536
Women	148.9^a^	9.5	159.8^b^	8.6	4.6	0.0351

SEM, standard error of the mean. *F* is the *F*-statistic, the ratio of the between group variance over the within group variance. Means have been adjusted based on the covariates. Model 1 adjusted for differences in: age, race, year of assessment, alcohol use, smoking status, and time spent in moderate to vigorous physical activity. Model 2 included the same covariates as model 1 and also total energy intake (kcal), and consumption (g per 1,000 kcal) of fiber, carbohydrate, protein, fat, sugar, and saturated fat. In model 2, the caffeine difference for men was borderline significant (*p* = 0.0536). There were 78 degrees of freedom in the denominator. For women, abdominal obesity was defined as waist circumferences >88 cm and for men >102 cm. Means on the same row with the same superscript letter are not significantly different.

Regression analysis showed that in U.S. men and women considered separately, energy intake (kcal) increased as body weight increased. In women, after adjusting for differences in age, race, year of assessment, alcohol use, smoking, and moderate to vigorous physical activity, for each 1 kg higher body weight, energy intake was 1.5 kcal higher, on average (F = 9.4, p <0.0029). In men, for each 1 kg higher body weight, energy intake was also 1.5 kcal higher, on average (F = 5.6, p <0.0206).

Table 5 displays the mean differences in energy intake (kcal) across tertiles of caffeine intake, after adjusting for differences in the covariates. For both men and women treated separately, the relationship was strong and dose-response. Specifically, adults in the high caffeine intake category consumed the most kcal, followed by the moderate caffeine intake group, and then the low caffeine intake group, that ingested the fewest kcal.

**Table 5:** Mean differences in energy intake (kcal) across tertiles of caffeine consumption, after adjusting for the covariates (*n* = 19,660).

	**Caffeine intake tertiles (mg)**							
	**Low**		**Moderate**		**High**			
**Outcome variable**	**Mean**	**±SEM**	**Mean**	**±SEM**	**Mean**	**±SEM**	***F***	***p*** **-value**
**Energy intake (kcal)**	
**Model 1**	
Men	2,285^a^	23	2,392^b^	21	2,604^c^	30	37.9	<0.0001
Women	1,712^a^	13	1,780^b^	16	1,894^c^	18	25.9	<0.0001
**Model 2**	
Men	2,275^a^	34	2,387^b^	33	2,590^c^	39	34.0	<0.0001
Women	1,738^a^	27	1,806^b^	26	1,919^c^	25	25.3	<0.0001

SEM, standard error of the mean. *F* is the *F*-statistic, the ratio of the between group variance over the within group variance. Caffeine intake tertiles were gender based. For women, low, moderate, and high caffeine amounts were 0–57.5, 58–165, and >165 mg, respectively. For men, the low, moderate, and high tertiles were 0–73.5, 74–218, and >218 mg, respectively. Means were adjusted based on the covariates. Model 1 included the demographic covariates: age, race, and year of assessment. Model 2 included the same covariates as model 1 plus body weight, alcohol intake, smoking status, and time spent in moderate to vigorous physical activity. There were 78 degrees of freedom in the denominator. Across each row, each mean differed significantly from each other mean. Means on the same row with the same superscript letter are not significantly different.

In summary, after adjusting for variability in all the covariates, age, race, year of assessment, alcohol consumption, smoking, moderate to vigorous physical activity, total energy consumption, and intake (g per 1,000 kcal) of carbohydrate, protein, fat, fiber, sugar, and saturated fat, there was a significant relationship between higher BMI and waist circumference levels and higher caffeine consumption in both men and women. The associations were linear in U.S. men and quadratic in U.S. women. The BMI and caffeine relationship was stronger in men than women. However, with waist circumferences divided into two categories, normal and abdominal obesity, the association was stronger in U.S. women than men. Finally, there was a strong, dose-response relationship between caffeine intake and energy (kcal) consumption with men and women treated separately. The higher the intake of energy (kcal), the greater the amount of caffeine consumed.

## Discussion

The present study asked the question, in the typical setting where adults dictate their own consumption of caffeine and food, do adults with excess body weight or excess abdominal fat use caffeine more or less than their peers? Four important findings were revealed: (1) the relationship between BMI and caffeine consumption was dose-response in U.S. men. In short, the relationship was linear. The higher the BMI, the greater the caffeine intake. (2) U.S. men with abdominal obesity consumed more caffeine than men with normal waist circumferences. (3) U.S. women with overweight consumed more caffeine than normal weight women and underweight women. However, the association was not dose-response. (4) U.S. women with abdominal obesity consumed more caffeine than women with normal waist circumferences.

The key findings suggest that U.S. adults with higher BMI levels and larger waists tend to consume more caffeine than their counterparts. There are multiple possible explanations for this relationship. First, the effect of caffeine on the body and mind is dose dependent. In a double-blind randomized experiment by Del Coso et al., (18) they found that blood pressure and heart rate increased in a “dose response manner” indicating that caffeine physically affected participants based on how much they consumed (page 4). Another study conducted by Nehlig and Boyet found that as the intake of caffeine increased in rodents, more brain regions were activated. Specifically, lower doses activated fewer regions, while higher doses resulted in broader brain activation (19).

### Therapeutic dose

Consuming a caffeine dose that results in the desired effect is important. A dose that is too low will be ineffective, while a dose that is excessive can lead to side effects. In a literature review by Nawrot et al., (20) the authors discuss how physiological responses to caffeine ingestion vary based on dosage, although there is individual variation. Their review highlighted cases where some have reported toxicity and death at doses of 6.5 g of caffeine while others have reported surviving after consuming 24 g. They concluded that daily ingestion of 500–600 mg of caffeine represents a substantial health risk and is outside the therapeutic range for most adults (20).

According to Guest et al., (21) a caffeine dose of 3–6 mg/kg of body weight was a therapeutic dose for improving exercise performance. Bougrine et al. (22) found that 6 mg/kg body weight improved athletic performance more effectively than 3 mg/kg, but 9 mg/kg produced side effects. Also, a meta-analysis by Grgic (23) showed that a dose as low as 0.9–2.0 mg/kg improved physical endurance and muscular strength. Finally, Filip-Stachnik et al. found that 3 and 6 mg/kg both caused a significant increase in muscular strength performance. However, the higher caffeine dose was significantly more effective than the lower dose (24). In general, these investigations indicate that weight-based dosing helps achieve optimal effectiveness. Therefore, it makes sense that in an unmanipulated, natural environment, which was the case in the present study, larger individuals consumed larger amounts of caffeine.

From a cognitive perspective, Smith et al. (25) reviewed the effects of caffeine on attention, vigilance, and reaction time. They concluded that 40–300 mg was a meaningful therapeutic range. Einother et al. (26) noted that 75–250 mg was an effective dose for simple and complex attention tasks. Finally, Nehlig (27) reviewed several investigations and concluded that a range of 100–400 mg of caffeine enhances reaction time, executive function, and memory.

### Appetite suppressant

Another reason heavier adults and those with greater abdominal adiposity might consume larger amounts of caffeine could be because many individuals with excessive weight would like to lose weight. Because caffeine acutely suppresses appetite, it is consistent that heavier adults would choose to consume more caffeine than their lighter counterparts to assist with weight management.

Several investigations have studied the appetite suppressing effects of caffeine. In a randomized controlled study by Gavrieli et al., normal weight and overweight/obese participants consumed a standard breakfast with 3 or 6 mg of caffeine per kg body weight. Three hours later, they consumed an *ad libitum* meal. The higher caffeine treatment reduced the energy consumed during the *ad libitum* meal and for the total day in the individuals with overweight/obese (28).

In another study conducted by Gavrieli et al., the researchers again found similar outcomes regarding caffeine as an appetite suppressant. They had 17 male participants consume water, 3 mg of caffeine, or 6 mg of caffeine in coffee. They found that those who ingested higher amounts of caffeine had their hunger suppressed for 150–180 mins compared to water (29). Additionally, a cross-over randomized investigation by Gkouskou et al. (30) found that coffee consumption was linked to a decrease in energy intake and appetite, especially for those who could quickly digest the caffeine.

### Reduced physical and mental fatigue

It is logical that heavier adults tend to be less fit and feel more tired than their counterparts. According to Wlodek et al., (31) heavier adults “experience fatigue and decreased physical endurance” (page 33). Caffeine is a stimulant drug that provides the consumer with an energy boost. Therefore, caffeine may be more attractive to heavier adults than their counterparts as a means of reducing fatigue.

Maqsood et al. (32) explain that caffeine has the potential to improve various cognitive and behavioral processes, such as increasing alertness and reducing feelings of fatigue. In support of this statement, Glade et al. conducted a detailed literature review on the effects of caffeine intake. He concluded that caffeine increases the amount of energy one has available which allows people to overcome physical and mental fatigue. He also stated that caffeine boosts focus and coordination (33). Additionally, Walton et al. (34) conducted a double-blind study to test caffeine's effect on self-sustained firing in the human brain and found that individuals who consumed caffeine had a higher occurrence of neuromuscular activity. In short, caffeine reduces physical and mental fatigue, which may be more appealing to those with overweight and obesity than others.

### Increased metabolism

Another possible explanation for the unmanipulated body size and caffeine intake association identified in the present investigation is because caffeine promotes the metabolism of body fat. In a study conducted by Acheson et al., (35) the researchers found that during steady conditions, caffeine consumption caused lipids to be processed twice as fast compared to no caffeine. In a study by Ding et al., (36) they gave caffeine to mice that were fed a high fat diet beforehand. The mice that consumed caffeine had significantly reduced liver fat. These findings suggest that adults with higher BMI and waist circumference levels may consume more caffeine than others for its potential fat-burning benefits.

### Inexpensive weight loss drug

A final, straightforward reason that heavier U.S. adults tend to consume more caffeine than their counterparts is that it is an inexpensive and commonly used drug that may help those with obesity reach a heathier weight. Kanj and Levine (37), in their review article, explained that many insurances do not cover weight loss drugs. Weight loss drugs can be extremely expensive (38). Meanwhile, caffeine is found in multiple foods and beverages, including soft drinks, tea, coffee, some medicines, energy drinks, and chocolate, and it is inexpensive.

Several investigations support the use of caffeine to help with weight control. In a prospective cohort study of three major investigations, researchers found that those who consumed unsweetened caffeinated coffee had an inverse relationship with gaining weight. The amount of weight gained over a 4-year period decreased by 0.12 kg per daily cup of coffee (39). Another study found that mice who were fed a high fat diet then caffeine ultimately developed reduced liver fat (36). Caffeine may be more appealing to heavier adults because it is a low-cost strategy that might help with attaining a healthier weight.

### Gender differences

The association between BMI and caffeine consumption was weaker in U.S. women than U.S. men, although the waist size and caffeine relationships were similar. One explanation for the difference could be hormone variations between the sexes causing individuals to feel the effects of caffeine differently. Temple and Ziegler conducted a double-blind, placebo-controlled study and found that more males than females told interviewers that they experienced and enjoyed the caffeine effect. When this was further examined, the researchers found that women reported these emotions like their male counterparts when estradiol levels were higher; however, steroid hormones had no impact on the male response (40).

A study conducted by Domaszewski came to similar conclusions as Temple et al. Men and women were given caffeine based on how much they consumed on a daily basis. Those who consumed low or moderate amounts of caffeine daily received 3 mg of caffeine for every kg of body weight. Those who consumed high amounts of caffeine daily received 6 mg of caffeine for every kg of body weight. They found that women reported feeling anxiety and worry three times more than men. Men reported feeling more energetic and cognitively functional than women. This study proposed that the differences in body composition between men and women could be one of the reasons why they feel and respond to caffeine ingestion differently (41).

### Side effects of caffeine

Caffeine consumption can have side effects, influenced by individual sensitivity, dosage, and hormonal variations. In a literature review by de Souza et al., researchers found that the most common side effect was irregular or rapid heartbeats that exceeded the normal range, especially those that consumed high amounts of caffeine (~6 mg/kg body weight). Other side effects included nervousness, headaches, and increased urination. Sleep was also affected as it took longer to fall asleep, total time sleeping decreased, and quality decreased (42). Another literature review conducted by Soós et al. (43) found similar results stating that those who consumed high amounts of caffeine (1,000 mg/day) had “toxic symptoms, restlessness, hyperactivity, headaches, nausea, dizziness, trembling, spasm, extrasystole, and tachycardia.”

However, the most significant side effect of caffeine is its addictiveness. When adults consume caffeine daily and then stop or significantly reduce their intake, they suffer from withdrawal. In a literature review by Juliano et al., they found 10 symptoms that were common in multiple studies. These were headache, fatigue, decreased energy/activeness, decreased alertness, drowsiness, decreased contentedness, depressed mood, difficulty concentrating, irritability, and foggy/not clearheaded (44). Caffeine addiction can have both physiological and psychological effects on the human body. When those with higher BMI and waist levels consume caffeine on a regular basis, they may continue in order to avoid the side effects associated with withdrawal.

### Energy intake and caffeine consumption

Energy expenditure is higher in larger adults. Therefore, under normal conditions, larger individuals consume more energy (kcal) than their counterparts. This was confirmed in the Results paragraph to the right of Table 2. In the present investigation, larger adults also consumed more caffeine than smaller adults (see Tables 3, 4). Given larger adults consumed more energy and more caffeine than smaller adults, it is consistent that adults who consumed more energy (kcal) also consumed more caffeine, as shown in Table 5. In this investigation, the association between energy intake (kcal) and caffeine intake was strong, positive, and dose-response. This may seem inconsistent with the literature because the literature indicates that caffeine is a metabolic stimulant and an appetite suppressant.

### Are the current findings inconsistent with the literature?

If regular caffeine ingestion reduces appetite, increases metabolism, and decreases fatigue, then why do adults with the highest caffeine consumption weigh significantly more than their counterparts? A close look might explain the apparent inconsistency. It seems that much of the apparent inconsistency is a result of a cross-sectional view vs. a prospective perspective.

The current study provides a cross-sectional view. The cross-sectional findings indicate that larger adults consume significantly more caffeine than smaller individuals. This makes sense for two reasons: (1) to be effective, a larger dose is needed for larger adults. (2) Many individuals with obesity want to lose weight. They want to have more energy and endurance. Consequently, they use caffeine to assist them. If caffeine is successful in helping those with obesity lose significant amounts of weight, then they may need less caffeine in the future because they are smaller, and the therapeutic dose for smaller individuals is lower.

The question of whether larger adults consume more caffeine than smaller individuals was answered by the present study using a cross-sectional research design. However, a carefully conducted prospective design will be needed to determine how body size and caffeine consumption interact and change over time.

### Summary

In general, the results suggest that U.S. men, and to a lesser extent U.S. women, consume larger amounts of caffeine if they are heavier and/or if they have larger waists than their counterparts. The explanation behind the association between body size and caffeine intake may be because heavier individuals typically need larger doses of caffeine to get the same treatment effects. Their therapeutic dose is higher. It could also be that caffeine is an appetite suppressant, which tends to assist with weight loss. Caffeine also helps with increased fat metabolism. Additionally, caffeine reduces fatigue, which is more common in adults with overweight or obesity. In short, the finding that heavier adults choose to consume larger amounts of caffeine than lighter individuals is consistent with the multiple perceived benefits larger adults hope to receive from consuming caffeine.

### Limitations and strengths

There were several limitations associated with this study. The first was that the NHANES design was cross-sectional, providing a snapshot view of the relationship between body size and use of caffeine. This limits the ability to establish causality between variables. Another limitation was that to obtain dietary information, NHANES used two 24-h dietary recall interviews. Since diet was self-reported, despite a standardized and precise protocol including food probes, there could be inaccuracies due to recall bias and misreporting. Lastly, while this study took age, sex, race, year of assessment, alcohol use, smoking, moderate to vigorous physical activity, and consumption of dietary fiber, carbohydrate, protein, fat, sugar, saturated fat, and total energy (kcal) into consideration, there are other covariates, unmeasured in this investigation, that could affect the relationship between BMI, waist circumference, and caffeine intake.

The present investigation also had multiple strong points. First, this study had a very large sample of almost 20,000 individuals randomly selected from the U.S. population. It was a diverse group representing all major races and ethnic groups and adults aged 20–75 years. Second, many covariates were controlled statistically to better isolate the relationship between BMI, waist circumference, and caffeine intake. Lastly, NHANES data was collected by trained professionals who used standardized practices and were evaluated regularly so the raw data was valid and reliable.

## Conclusions

The present study examined the relationships between BMI, waist circumference, and caffeine intake and found that U.S. men who were heavier and/or had larger waists used more caffeine than leaner men. In men, the association was dose-response. On the other hand, in U.S. women, the relationship between BMI and caffeine intake was significant, but not linear. Specifically, the association was quadratic, with caffeine levels decreasing toward the upper values of BMI. With waist size divided into two categories, normal and abdominal obesity, the waist circumference and caffeine intake relationship was stronger in women than men. In general, it appears that caffeine has multiple properties that appeal to adults with higher BMI and waist circumference levels. Additional research is needed to further clarify why larger men and women choose to consume more caffeine than their counterparts. There are multiple reasons that could account for the associations.

## Data Availability

Publicly available datasets were analyzed in this study. This data can be found here: NHANES questionnaires, datasets, and related documentation at https://wwwn.cdc.gov/nchs/nhanes/Default.aspx.
